# Particle Size Analysis in Aerosol-Generating Dental Procedures Using Laser Diffraction Technique

**DOI:** 10.3389/froh.2022.804314

**Published:** 2022-02-11

**Authors:** Kaoru Onoyama, Shohei Matsui, Mariko Kikuchi, Daisuke Sato, Haruka Fukamachi, Miki Kadena, Takahiro Funatsu, Yasubumi Maruoka, Kazuyoshi Baba, Kotaro Maki, Hirotaka Kuwata

**Affiliations:** ^1^Division of Community-Based Comprehensive Dentistry, Department of Special Needs Dentistry, Faculty of Dentistry, Showa University, Tokyo, Japan; ^2^Department of Implant Dentistry, Faculty of Dentistry, Showa University, Tokyo, Japan; ^3^Department of Oral Microbiology and Immunology, Faculty of Dentistry, Showa University, Tokyo, Japan; ^4^Division of Dentistry for Persons With Disabilities, Department of Special Needs Dentistry, Faculty of Dentistry, Showa University, Tokyo, Japan; ^5^Department of Pediatric Dentistry, Faculty of Dentistry, Showa University, Tokyo, Japan; ^6^Department of Prosthodontics, Faculty of Dentistry, Showa University, Tokyo, Japan; ^7^Department of Orthodontics, Faculty of Dentistry, Showa University, Tokyo, Japan

**Keywords:** COVID-19, infection control, dental public health, airborne transmission, aerosol generating dental procedure, transmission based precautions, laser diffraction analysis, particle size analysis

## Abstract

The global outbreak of coronavirus disease 2019 (COVID-19) has raised concerns about the risk of airborne infection during dental treatment. Aerosol-generating dental procedures (AGDP) produce droplets and aerosols, but the details of the risks of COVID-19 transmission in AGDP are not well-understood. By discriminating between droplets and aerosols, we devised a method to measure particle size using laser diffraction analysis and evaluated aerosols generated from dental devices for providing a basis for proper infection control procedures. The droplets and aerosols generated from dental devices were characterized by multimodal properties and a wide range of droplet sizes, with the majority of droplets larger than 50 μm. AGDP emitted few aerosols smaller than 5 μm, which are of concern for pulmonary infections due to airborne transmission. In addition, the use of extraoral suction was found to prevent the spread of aerosols from high-speed dental engines. This study suggests that the risk of aerosol infections is considerably limited in regular dental practice and that current standard precautions, such as mainly focusing on protection against droplet and contact infections, are sufficient. While several cases of airborne transmission of COVID-19 in general clinics and emergency hospitals have been reported, cluster outbreaks in dental clinics have not yet been reported, which may indicate that AGDP does not pose a significant threat in contributing to the spread of SARS-CoV-2.

## Introduction

The Coronavirus disease 2019 (COVID-19) caused by severe acute respiratory syndrome coronavirus 2 (SARS-CoV-2) has occasioned a serious pandemic, radically changing the life of people. Despite the accumulation of a considerable amount of useful information, including epidemiological analyses, genetic studies, and SARS-CoV-2 virulence factors [1, 2] consensus on the transmission route of COVID-19 has not been firmly established. According to a WHO report, the main transmission routes of SARS-CoV-2 are contact and droplet transmission, but the airborne transmission of virus particles can occur during aerosol-producing medical procedures in the hospital, especially in poorly ventilated indoor environments [3]. Indeed, a community of aerosol researchers is actively investigating the risks and ubiquity of airborne infections [4]. There has been intense debate over the prevention of SARS-CoV-2 infection, and further data on infection routes are still necessary. Current evidence for the transmission of SARS-CoV-2 includes the presence of the viral genome in air samples in hospital environments [5, 6]. Besides medical settings, airborne infection also occurs in public transport [7]. Thus, it is difficult to completely deny the possibility of airborne transmission of COVID-19. While cluster infections have not been reported in dentistry, potential risks exist, especially when a patient is suspected of being infected with SARS-CoV-2 [8, 9]. Droplet and airborne transmissions in dentistry were previously recognized in the 2003 outbreak which was a viral respiratory disease caused by a SARS-associated coronavirus [10], but the COVID-19 outbreak has reawakened the threat of aerosols [11, 12]. It is thought that regular dental practice can be a source of aerosols, and dental clinics are high-risk sites for droplet and airborne infections [13]. Droplet and aerosol generation in dental practice has long been a traditional research agenda [14, 15]. Droplet and aerosol size are significant in assessing the risk of droplet and airborne infections, and more studies on how and to what extent aerosols are produced from aerosol-generating dental procedures (AGDPs) are required to verify infectivity in confined areas [16]. Previous studies on droplet and aerosol size distinguish “aerosols” from “droplets” at a threshold of 5 μm; “droplets” larger than 5 μm can cause droplet infections but fall quickly under gravity to within 1 meter of the source. “Aerosols” (or droplet nuclei) remain in the air for a long time and can cause respiratory infection in the alveoli [9, 17]. To quantitatively assess the risk of respiratory infections from aerosols, measuring particle size distribution (PSD) provides a solid scientific basis [18, 19]. Among several techniques for measuring PSD, laser diffraction analysis allows precise, real-time measurement of a wide range of particle sizes (0.1–1,000 μm) along with high time resolution. The technique has been successfully used in studies to quantify the size of aerosols generated from coughs [20] and nasal sprays [21].

In this study, we accurately and quantitatively investigated PSD from various dental devices using the cylinder system combined with laser diffraction analysis. Since the use of extraoral suction is a conventional infection prevention procedure in dentistry [22, 23], we also tested the efficacy of suctioning aerosols with an extraoral suction, to explore the effective location of extraoral suction with regard to aerosol sources. This research aims to provide not only necessary information for daily dental practice but also concrete knowledge for the establishment of future standard preventive methods in dentistry by public organizations.

## Materials and Methods

### Laser Diffraction System Setting

**Figure 1:**
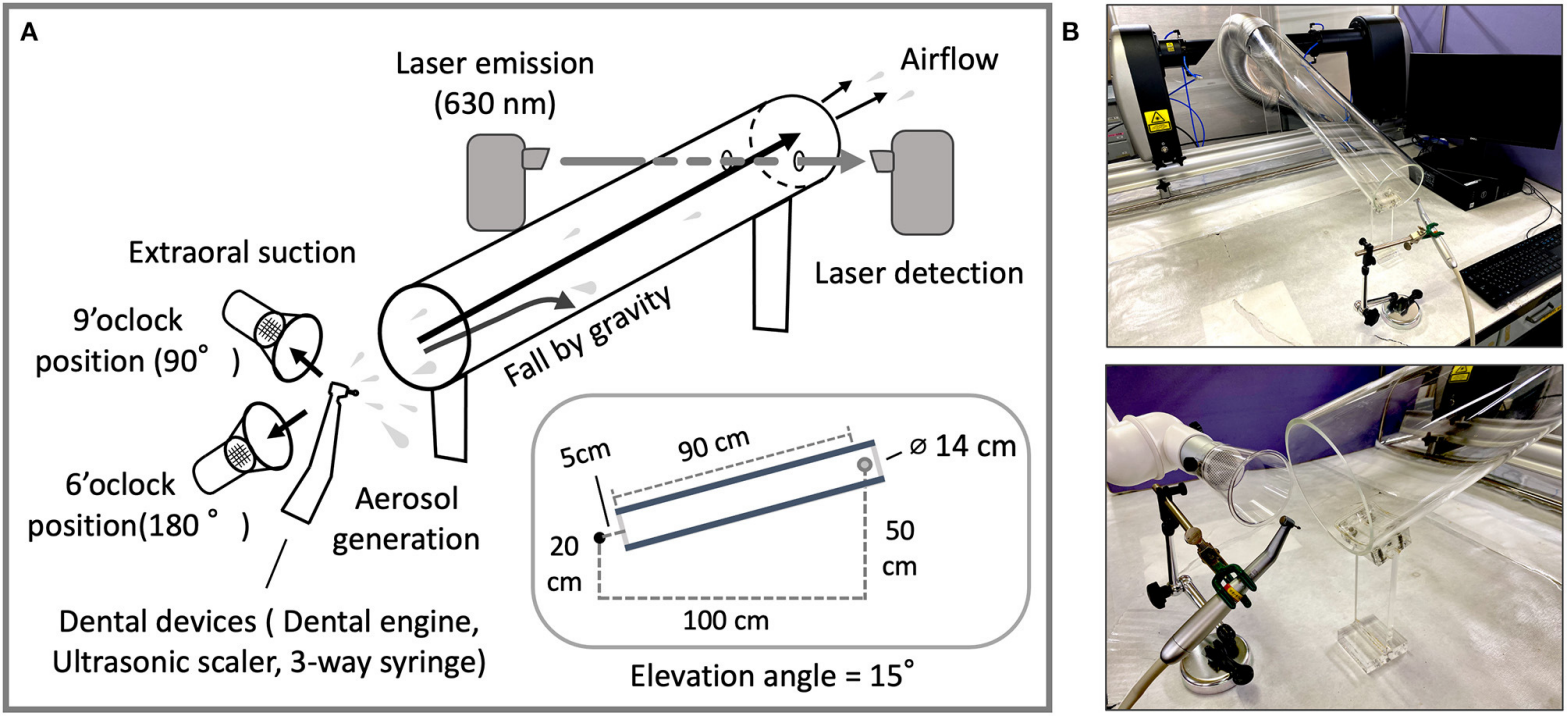
**(A)** Through-cylinder system; layout and schematic of the cylinder and laser detection device for aerosol measurement. The acrylic cylinder (20 cm in diameter and 90 cm in length) is set at an angle of 15 degrees. Aerosols from devices were transported by airflow (0.8 m/sec) to the laser position and particle size data was taken at a real-time speed (1 Hz) for 25 seconds. **(B)** The relationship between the position of the aerosol source and the aerosol measurement device. Dental devices were fixed to the column stand (upper). As an example of use, the extraoral suction is placed at the 9 o'clock position to the left of the dental instrument with the direction of aerosol ejection at the 12 o'clock position (lower). The system was covered during measurement to prevent excess airflow from the exterior by a vinyl sheet and the safety cabinet.

All experiments were conducted at room temperature (25°C) and ~50% RH humidity, maintaining the temperature and humidity of the laboratory from spring to fall of 2021. Data acquisition and analysis were conducted in the Hatanodai Center Laboratory of Showa University. In this experimental study, we designed a measurement system that reduces the number of large droplets that fall naturally by passing them through a cylinder, thus more accurately measuring the size of aerosols transported by airflow. The evaluations of droplet size were performed using a Spraytec real-time spray particle analyzer (STP5321; Malvern Panalytical, Worcestershire, UK), with a focal lens of 300 mm and prepared to measure droplets from 0.10 to 1,000 μm. Scan time (approximately 5 s) was determined manually between 75 and 95% by transmittance reduction. Measurements were performed with a laser of 630 nm wavelength of He-Ne laser (4 mW). Water was purified by reverse osmosis membrane (Elix-UV3; Merk Millipore, MA, USA) and used for all experiments. To verify the effect of aerosols passing through the cylinder, the acrylic cylinder was assembled manually. The acrylic cylinder is 90 cm long and 14 cm in diameter. The distance between the aerosol source and the laser measurement position is 100 cm. The site of laser passage in the acrylic cylinder was perforated. The aerosol source and the laser measurement point are at a height of 20 and 50 cm from the bench, respectively. The elevation angle of the acrylic cylinder is 15° (Figure 1, side view shown in inset). The airflow was generated using a vacuum cleaner. The air velocity was 0.8 ± 0.0 m/s at the position of aerosol generation measured by the vane method. The sets of airflow rates were designated in 10 levels ranging from 1 as low to 10 as strong. Each air velocity was 3.7 ± 0.5 and 6.7 ± 0.5 m/s, respectively. The extraoral suction (EVA-Q; BSA - Sakurai, Nagoya, Japan) was placed at a distance of 5 cm and used for the air aspiration experiment.

### Aerosol Generator

All dental devices used in this experiment were part of the portable dental unit (Daisy OPU-D2; Osada electric, Tokyo, Japan). Rotary dental instrument (WG-99LT, Osada electric) equipped with pear-shaped diamond bur (F0-25, Mani Inc., Tochigi, Japan), an ultrasonic scaler (ENAC-OE-11L, Osada electric), and a 3-way syringe (MS-F, Osada electric Inc) were attached as equipment. Nebulization was performed using compressor-type nebulizers (NE-C28; Omron, Kyoto, Japan). The ultrasonic home-use humidifier (DH-HB350; Ohtake, Fukushima, Japan) was used. Before data acquisition, the dental devices were started, and after confirming stable aerosol generation for a minute, aerosol was supplied to the cylinder, and after confirming stable aerosol passage for a minute, the laser was emitted, and data were continuously acquired for about 30 s at intervals of a minute.

### Statistical Analysis

Statistical analysis was performed using the non-parametric Mann-Whitney U test by JASP ver. 0.16 (JASP Team, Amsterdam, Netherland). For statistical comparison of droplet and aerosol diameters, the indices of Dv10, Dv50, Dv90, and Span were calculated by Spraytec software ver. 4.00 (Malvern Panalytical). For the explanation for each value, Dv10, Dv50, and Dv90 represent the combined particle volume of the 10, 50, and 90% of the cumulative volume, respectively, in the particle size distribution of droplet particles. Span was calculated as (Dv90 – Dv10)/Dv50.

## Results

### Development of Droplet and Aerosol Measurement Device With the Integrated Cylinder

In preliminary experiments, we found that droplets and aerosols from dental devices varied widely in size. Hence, concerns arise that, theoretically, larger particles inhibit the accurate measurement of smaller-sized particles. In order to accurately and broadly measure the size of particles from dental devices regardless of the size of water droplet particles, we assembled an airborne aerosol measurement device that combines an acrylic cylinder with a laser diffraction particle size analyzer, by separating small aerosols from large droplet particles (Figure 1). Since large droplets not carried by the air stream hit the cylinder because of their weight and do not reach the laser position, more accurate measurement of smaller-sized aerosols can be performed.

### Evaluation of Droplet and Aerosol Generated From Various Dental Devices by the Through-Cylinder System

**Figure 2:**
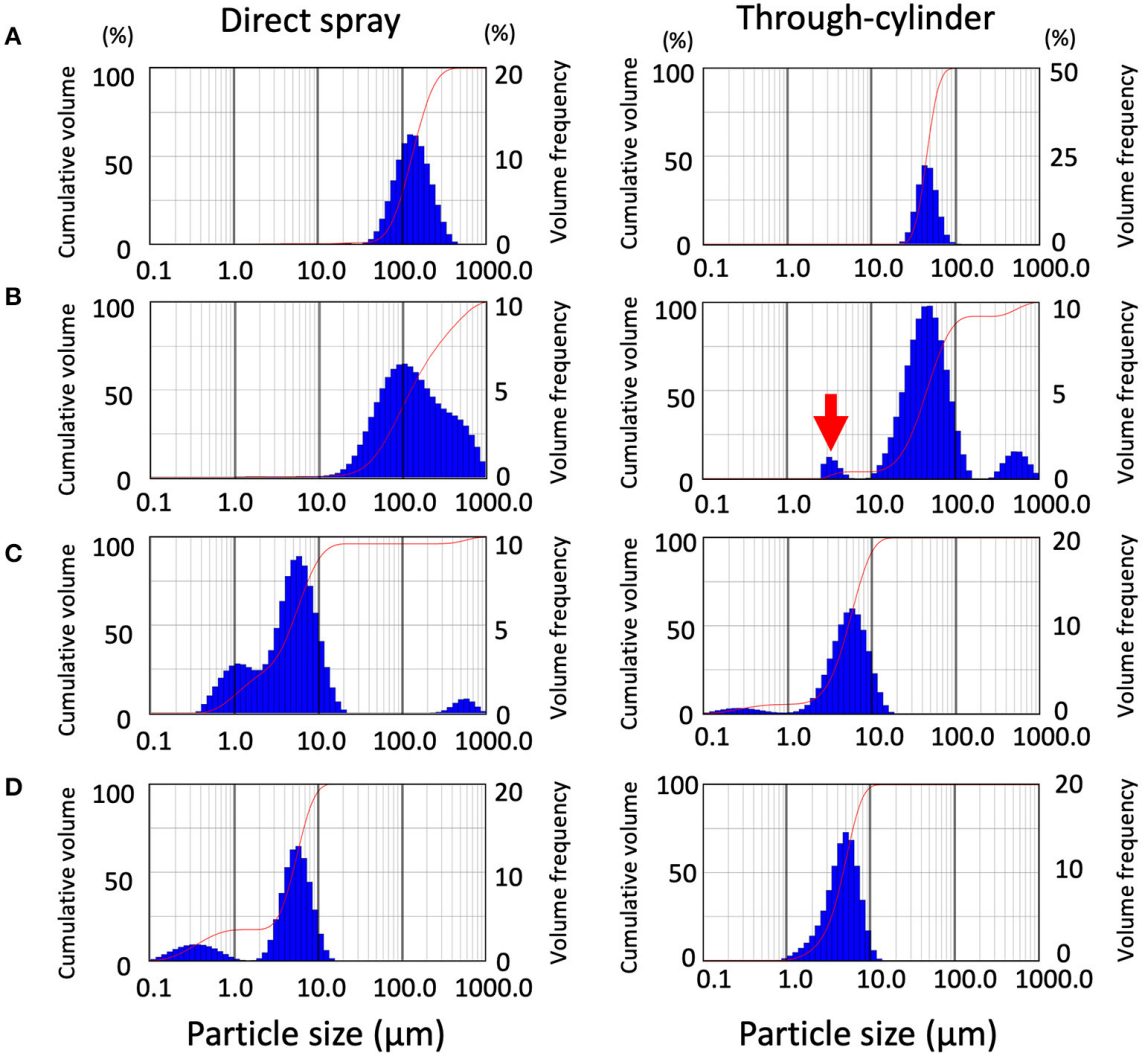
Distribution patterns of droplet size emitted from the devices and validation of the removal effect of the cylinder on large droplets. In each data set, data from direct spraying (left), and through-cylinder (right) are shown separately. Droplet size distribution (DSD) histogram graph emitted from ultrasonic scaler **(A)**, 3-way syringe **(B)**, nebulizer **(C)**, humidifier **(D)**. The curve in the graphs represent the droplet cumulative volume frequency and the percentage of a particular size of the droplet. Each experiment was performed independently at least four times, and the results of one representative experiment are shown.

First, an ultrasonic scaler and a 3-way syringe were used as dental devices. Besides the dental devices, medical nebulizers and home-use humidifiers were similarly examined for comparison. Using laser diffraction which is specifically designed for splatter characterization in the air, the size and distribution of droplets and aerosols were examined. First, we looked at the difference in distribution patterns of droplets and aerosols using the methods of direct spraying and spraying through the cylinder. The median droplet size generated by the ultrasonic scaler was 142.3 ± 9.33 μm as Dv50 for direct spraying, and 43.16 ± 1.73 μm as Dv50 for through-cylinder. In through-cylinder spraying, droplets ranging between 100 to 1,000 μm were rarely observed (Figure 2A and Table 1). This suggested that passage through the cylinder removed large droplets. The smallest droplets generated by the ultrasonic scaler were ~50 μm, which is larger than the typical 5 μm of aerosols that cause airborne transmission. We also examined droplets and aerosols generated from a 3-way syringe. Median droplet diameter was 147.42 ± 10.76 μm as Dv50 when measured by direct spraying, and 42.03 ± 10.76 μm as Dv50 through the cylinder (Table 1). In addition, aerosols of 2–5 μm diameter were observed only through the cylinder (Figure 2B right, red arrow). It is thought that this is the effect of through-cylinder measurement which eliminates large droplets and highlights the presence of small aerosols. Statistical comparison of the volume percentile showed that droplets and aerosols from the ultrasonic scaler and 3-way syringe had significantly smaller diameters when passed through the cylinder (Table 1). These results show that the through-cylinder method can more clearly measure aerosol diameter, which is difficult to measure when there are mixed droplets and aerosols of various sizes.

**Table 1:** Numerical parameters of droplet size distribution in aerosols between direct spraying group and through-cylinder group.

		**Dv10 [μm]**	**Dv50 [μm]**	**Dv90 [μm]**	**Span**
Ultrasonic	Direct	79.07 ± 6.84	142.30 ± 9.33	254.52 ± 12.86	1.24 ± 0.05
	Cylinder	31.57 ± 0.67^*^	43.16 ± 1.73^*^	58.80 ± 4.06^**^	0.63 ± 0.07^*^
3-way syringe	Direct	31.75 ± 1.28	147.42 ± 10.76	561.40 ± 31.79	3.60 ± 0.07
	Cylinder	17.75 ± 1.77^**^	42.03 ± 10.76^**^	110.35 ± 6.04^**^	2.22 ± 0.29^**^
Nebulizer	Direct	1.11 ± 0.11	4.96 ± 0.12	11.48 ± 0.38	2.08 ± 0.04
	Cylinder	2.94 ± 0.43^**^	5.47 ± 0.17^**^	9.20 ± 0.50^**^	1.15 ± 0.17^**^
Humidifier	Direct	0.37 ± 0.03	4.81 ± 0.18	8.45 ± 0.22	1.68 ± 0.03
	Cylinder	2.38 ± 0.01^*^	4.51 ± 0.13 ^ns^	7.34 ± 0.26^**^	1.10 ± 0.03^**^

*Each experiment was performed independently at least four times, and the results of one representative experiment are shown. For the quantitative analysis of the particle size distributions measured by laser diffraction in this study, the Dv10, Dv50, Dv90, and Span factors (each of which indicates the 10, 50 90% of the cumulative volume of the particles and particle size uniformity) were used. Data are represented as mean ± standard deviation*.

* *p < 0.01*,

** *p < 0.05*,

ns. *not significant, compared with each data of direct measurement (Mann-Whitney U-test)*.

Next, droplets and aerosol generation by the medical-use nebulizer and the home-use humidifier were measured using the same method. A nebulizer atomizes drugs to generate small and uniform size aerosols [24, 25], for example in asthma medications, to efficiently deliver to the bronchi and the alveoli in-depth [26]. However, because of this feature, nebulizers have been temporarily banned in medical institutions such as otorhinolaryngology clinics due to concerns about the risk of COVID-19 transmission [27]. We measured aerosols generated by nebulizers, both directly and via cylinders, ranging from 1 to 10 μm (Figure 2C). Furthermore, large droplets of 100 to 1,000 μm were only detected by direct measurement and not through the cylinder. Next, we also examined aerosols from a home-use humidifier. Results showed that they produced smaller-sized aerosols than dental devices and nebulizers, emitting two types of aerosols with water droplet diameters between 0.2 and 5 μm (Figure 2D). Previous reports have also shown that ultrasonic humidifiers produce aerosols as small as 1–10 μm [28], and small aerosols can remain airborne for a long time. In the nebulizer and humidifier measurement, the change of particle size in the through-cylinder method was not so obvious in the comparison of Dv50 and Dv90 of direct spraying and through-cylinder (Table 1).

### Aerosol Generation From Rotary Cutting Instruments and the Effect of Rotation Speed

**Figure 3:**
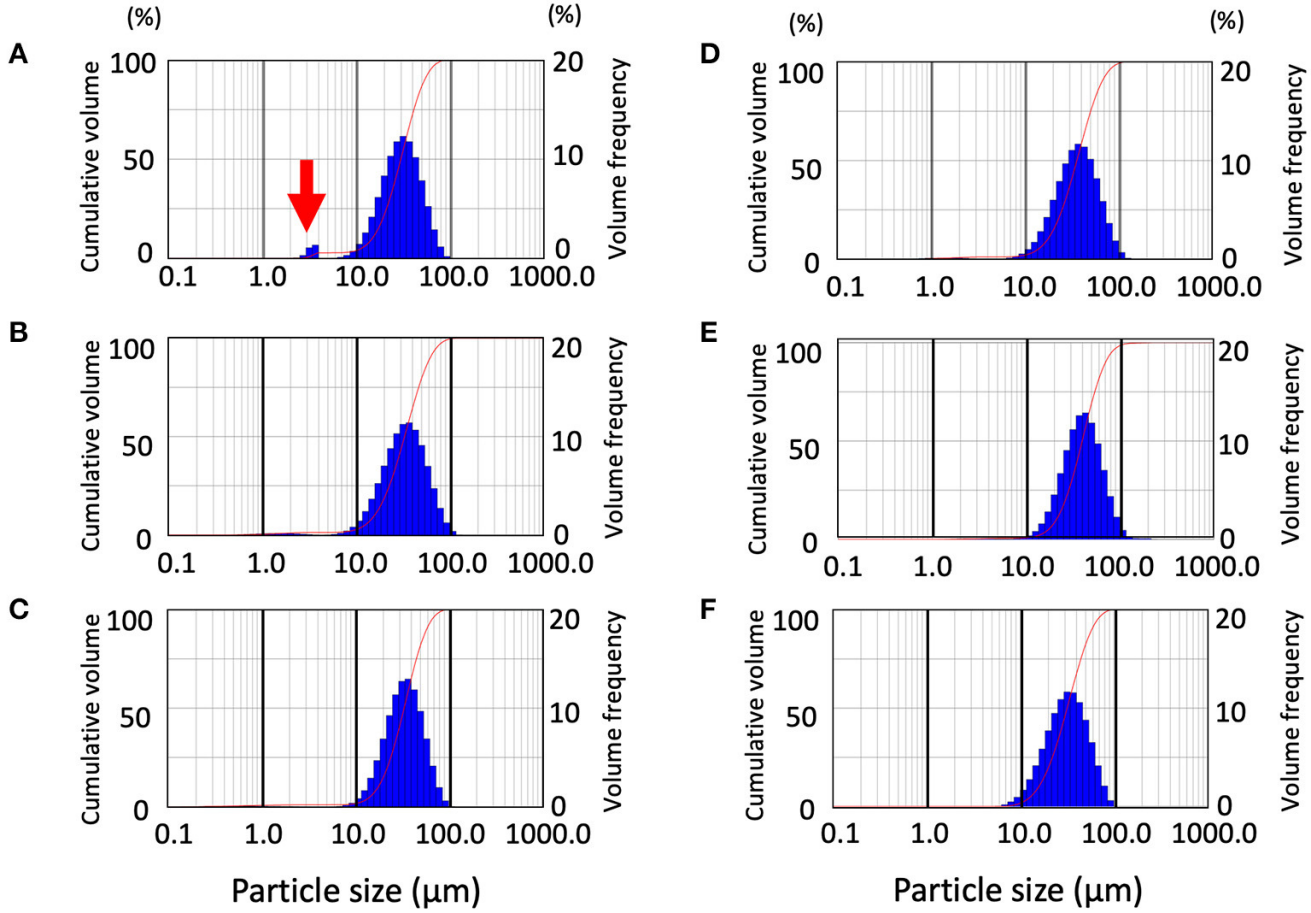
Distribution pattern of droplet size emitted from the dental engine at various speeds and validation of the effect of extraoral suction. Data from the through-cylinder is shown. Droplet size distribution (DSD) histogram graph emitted from the dental engine at the speed of 200,000 rpm **(A)**, 100,000 rpm **(B)**, 5,000 rpm **(C)**. Distribution pattern of droplet size emitted from the dental engine with extraoral suction by low set with 180 degrees, strong set with 180 degrees, and strong set with 90 degrees (**D–F**, respectively). The curves in the graphs represent the droplet cumulative volume frequency and the percentage of a particular size of the droplet. Each experiment was performed independently at least four times, and the results of one representative experiment are shown.

We measured droplets and aerosols generated by the rotary cutting dental instrument, at maximum speed and compared direct spraying and the through-cylinder system. Results showed that water droplets of 100–1,000 μm in size were removed by the through-cylinder system, which was consistent with results comparing ultrasonic scalers and 3-way syringes (Supplementary Figure). Then, aerosol generation from a dental engine was examined with the through-cylinder method. The dental engine tested in the experiment is available at speeds ranging from 5,000 to 200,000 rpm. At the highest speed of 200,000 rpm, the average Dv10 was 15.74 ± 0.64 μm (Figure 3A and Table 2). By contrast, at 5,000 rpm, Dv10 was 17.18 ± 0.28 μm, and at 10,000 rpm, Dv10 was 16.49 ± 0.72 μm), which was significantly larger than the Dv10 at 200,000 rpm (Table 2). The mean Dv50, Dv90, and Span did not change for each group. Of particular note: at 200,000 rpm, aerosols of about 3–5 μm were generated (Figure 3A, red arrow). Droplet diameters emitted from the 5,000 and 100,000 rpm rotations ranged from 10 to 100 μm and there was no significant change in the volume percentiles of the particle between the 5,000 and 100,000 rpm groups (Figures 3B,C, and Table 2). Consequently, the dental engine produced aerosols potentially causing airborne transmission mainly during use at maximum speed, such as 200,000 rpm and rarely at lower speeds. Thus, even at the highest-speed rotation, the frequency of aerosol formation was low and relatively less than from nebulizers and home humidifiers.

**Table 2:** Numerical parameters of the droplet size distribution in the aerosol from the dental engine at the various speed and conditions of the extraoral suction through-cylinder group.

**Speed (rpm)**	**Extraoral suction**		**Dv10 [μm]**	**Dv50 [μm]**	**Dv90 [μm]**	**Span**
	**Level**	**Direction**				
200,000	Off	**–**	15.74 ± 0.64	31.40 ± 1.48	56.09 ± 3.06	1.28 ± 0.03
100,000	Off	**–**	16.49 ± 0.72^ns^	33.47 ± 1.04^ns^	61.39 ± 1.42^ns^	1.34 ± 0.04^ns^
5,000	Off	**–**	17.18 ± 0.28^*^	33.63 ± 0.44^ns^	60.01 ± 1.51^ns^	1.27 ± 0.04^ns^
200,000	Low	180°	17.77 ± 0.41^*^	36.05 ± 0.37^*^	66.75 ± 0.66^*^	1.36 ± 0.01^*^
	Strong	180°	19.68 ± 1.48^*^	36.89 ± 2.59^*^	64.92 ± 4.83^*^	1.23 ± 0.01^ns^
	Strong	90°	15.94 ± 2.13^ns^	30.00 ± 4.40^ns^	46.07 ± 9.93^ns^	1.20 ± 0.11^ns^

*Each experiment was performed independently at least three times, and the results of one representative experiment are shown. The Dv10, Dv50, Dv90, and Span factors, each of which indicates the 10, 50 90% of the cumulative volume of the particles and particle size uniformity, were used. Data are represented as mean ± standard deviation*.

* *p < 0.05*,

n.s. *, not significant, compared with the data of 200,000 rpm and off-suction (Mann-Whitney U-test)*.

### Effect of Extraoral Suction on Aerosol Elimination

Next, to verify the effect of aerosol removal by extraoral suction, aerosols emitted from a dental engine at 200,000 rpm were aspirated by an extraoral suction, and the removal efficiency was analyzed by the through-cylinder system. Two sets of suction strength and two patterns of pointing angles were examined, and the data were then compared by the laser diffraction method after passage in the cylinder. First, we examined the aerosol removal effect of an extraoral suction directed from the back. Comparing suction strength under every condition of the extraoral suction, even at the lowest setting, all volume percentiles were significantly larger than the control data without extraoral suction, indicating that small aerosols were removed by the suction (Figures 3D,E and Table 2). When suction strength was increased to the maximum, each volume percentile was even larger, indicating that small aerosols were further aspirated (Table 2). Using medium suction strength, the effect was an intermediate value, indicating that removal depends on suction strength (Data not shown). Next, we examined the aerosol removal effect of an extraoral suction directed from the lateral side. Although small aerosols (3–5 μm diameter) were aspirated, neither volume percentile was significantly different from the control without suction (Figure 3F and Table 2). The result suggests that extraoral suction is highly effective in eliminating aerosols, however, it also suggests that more consideration should be paid to the orientation of the suction to the aerosol source.

## Discussion

The purpose of our experiment was to verify as accurately as possible how large and how many aerosols are generated by dental devices at AGDPs. We measured and quantitatively validated the diameters of aerosols and droplets generated by three dental devices: an ultrasonic scaler, a 3-way syringe, and a dental engine. The results show that all devices produce only small amounts of aerosols, and the potential risk for respiratory infections was low. Several previous reports suggest that SARS-CoV-2 can be transmitted by aerosols over short distances in confined areas [4–7, 29], however, the importance of airborne infection in the global pandemic is not conclusive [30]. The reports have indicated that the viral genome has been detected in air samples from hospital rooms and other enclosed spaces, but secondary infections to health care workers and others have not occurred when medical treatment was conducted without any airborne precautions [31]. Even before the COVID-19 pandemic, standard precautions were thoroughly followed in dental clinics. Although many water droplets and aerosols appear to be dispersed in dental practices, fortunately, no cases of transmission by aerosols have been identified in these environments [16, 31]. This suggests that considering the use of individual protection equipment such as filtering facepiece respirators and face shields, the airborne infection may not be a significant concern in dental practice.

Since the outbreak of the COVID-19 pandemic, a variety of measurement methods have been employed to evaluate the dispersion of droplets and aerosols from dental instruments commonly used in dental procedures.

The aerosol measurement method, combining laser diffraction analysis and the through-cylinder system, was effective in obtaining accurate measurements of aerosols from 1 to 10 μm in diameter, since large droplets of 100 to 10,000 μm, which hinder measurement, can be eliminated when measuring droplets and aerosols of various sizes. It is reported that the large droplets causing an infection rarely travel through the air for more than a 1-meter distance [9]. Our data is also consistent, with larger droplets being decreased via the cylinder. Thus, using this laser diffraction analysis method, we found that hazardous aerosols generated from dental devices are considerably limited. It is important to note that middle size droplets as large as 50 μm were generated from the dental devices and carried by airflow against gravity over a 1-meter distance. A further experiment will be considered in the future to determine how far middle-size droplets can travel. Sergis et al. [32] examined aerosol generation from a rotating dental instrument by a combination of capturing blue light illumination and an optical particle sizer and they showed that the most generation occurred at high rotation speed (180,000 rpm) and that a threshold for rotation in atomization existed between 80,000 and 100,000 rpm. Our data showed that most of the emitted droplets were 10–100 μm in size, regardless of the rotation speed, but Sergis et al.‘s report did not evaluate data in the range of 5–100 μm due to the limitation of their measurement method. Another paper [33], aimed at determining the mechanism of aerosol generation and the most effective inhibition method of aerosols by an extraoral suction system, also measured small size aerosols ranging between 1 nm and 1 μm generated from a high-speed turbine and which were detected using a scanning spectrometer in a typical clinical setting. The size of aerosols that are most effectively removed by extraoral suction is in the 1–10 μm range, which is not included in their measurement range. Furthermore, an additional group [34] used optical flow tracking to measure droplet size (measurement range 5–300 μm) from an ultrasonic scaler in an oral cavity model simulating a clinical environment, while yet another group [35] used a light-scattering airborne counter to validate the effect of aerosol aspiration by extraoral suction. This latter paper showed that extraoral suctions could aspirate aerosols, but there were no differences in aspiration effectiveness by size. On the other hand, our results showed that extraoral suction was effective in aspirating aerosols with a size in the 1–10 μm range, but had less effect in aspirating large droplets over 10 μm. These differences may be due to the advantage of our using the laser diffraction method. Thus, the measurement methods in all papers are different, and their interpretation of results also differed somewhat from each other. The lack of uniformity in the measurement methods of water droplet diameter has a significant effect on the interpretation of research data, therefore, a standard should be established in the future. The strength of our study is that we used laser diffraction which has a broader range to measure PSD.

In considering the transmission of pathogens such as viruses in the respiratory tract, diameters of aerosols and droplets are the most critical factor [36, 37]. Theoretically, the size of aerosol particles that can cause airborne infection is thought to be <5 μm, and interestingly the study at the Wuhan hospital showed that SARS-CoV-2 virus particles were mainly detected in aerosols in two peaks (0.25–1.0 and 2.5–5.0 μm), indicating that aerosols containing virus have bimodal characteristics [5]. From this paper, it is evident that the size of submicron aerosols containing viruses has aerodynamically variable characteristics, and as a result, the size of the particles decreases with environmental factors such as airflow and low humidity. Our study is based on the idea that more accurate aerosol measurements need to eliminate unpredictable factors such as vertical airflow, hence we performed aerosol measurements in the laboratory rather than in the dental office. Another feature revealed by the laser diffraction method is the multimodality of the particle size peaks emitted by AGDPs. Droplets and aerosols emitted from dental equipment, as well as medical nebulizers and home humidifiers, were shown to be composed of multiple sizes. The presence of multiple-sized aerosols makes the assessment of airborne generation far more complicated. This fact has not been clarified in previous papers and our study is the first to demonstrate this.

In conjunction with previous literature [38], it is reasonable to assume that because of their size, most aerosols produced by dental equipment do not directly reach the deep lung and the risk of severe lung infection is minimal. In addition to the particle size, numerous other factors in the oral cavity are thought to influence infection efficiency. For example, the presence of components such as mucosal-specific IgA antibodies, lactoferrin, and lysozyme inhibit viral infection. On the other hand, it has been shown that viral entry factors such as ACE2 and TMPRSS members were broadly expressed in oral epithelial cells, which may increase the chance of viral infection [39]. In addition to biological factors, the properties of the aerosols may differ depending on the type of dental engine and the type of bur. For a more comprehensive risk assessment, the study of AGDPS in dentistry needs to be approached from a broader perspective, integrating medicine, engineering, and environmental science. It is expected that more research will be conducted in the future [40]. The efficiency of infection seems to be determined by the sum of various factors, thus the study limitation is that the measurement of aerosol data from instruments in the actual clinical settings used in dental practice was not included.

## Conclusion

The generation of aerosols smaller than 5 μm, which is a source of concern in airborne transmission, was fairly low in dental treatment. To date, even in public medical institutions, there are few clear evidence-associated cases of SARS-CoV-2 infection spreading by airborne transmission. Thus, the conclusion to be drawn is that fundamental infection control in dental practice should focus on droplet and contact infections, and less on airborne infections.

## Data Availability Statement

The raw data supporting the conclusions of this article will be made available by the authors, without undue reservation.

## Author Contributions

KO and SM contributed to experiment design, data acquisition, analysis, interpretation, and critically revised the manuscript. MK contributed to conception and experiment design, contributed to data acquisition, analysis, interpretation, and drafted the manuscript. HF contributed to analysis and interpretation, drafted the manuscript, and critically revised the manuscript. MK and TF contributed to conception, design, analysis, interpretation, and critically revised the manuscript. DS, YM, KB, and KM contributed to conception, design, interpretation, and critically revised the manuscript. HK contributed to conception, design, data acquisition, analysis, interpretation, and drafted the manuscript. All authors gave their final approval and agreed to be accountable for all aspects of work ensuring integrity and accuracy.

## Funding

We received the Special Research Funding of Showa University.

## Conflict of Interest

The authors declare that the research was conducted in the absence of any commercial or financial relationships that could be construed as a potential conflict of interest.

## Publisher's Note

All claims expressed in this article are solely those of the authors and do not necessarily represent those of their affiliated organizations, or those of the publisher, the editors and the reviewers. Any product that may be evaluated in this article, or claim that may be made by its manufacturer, is not guaranteed or endorsed by the publisher.

## Abbreviations

COVID-19: Coronavirus disease 2019
SARS-CoV-2: Severe acute respiratory syndrome coronavirus 2
AGDP: Aerosol generating dental procedure
PSD: Particle size distribution.
