# Supplementary material for: Particle Size Analysis in Aerosol-Generating Dental Procedures Using Laser Diffraction Technique
Source: Front Oral Health. 2022 Feb 11;3:804314. doi: 10.3389/froh.2022.804314 (PMC8873144; doi:10.3389/froh.2022.804314)
Supplement: Supplementary file 1 [file Data_Sheet_1.DOCX]

**Supplementals**

**Particle size analysis in aerosol-generating dental procedures using laser diffraction technique.**

Kaoru Onoyama^1*^, Shohei Matsui^1*^, Mariko Kikuchi^1^, Daisuke Sato^2^,

Haruka Fukamachi^3^, Miki Kadena^4^, Takahiro Funatsu^4 5^, Yasubumi Maruoka^1^, Kazuyoshi Baba^6^, Kotaro Maki^7^, Hirotaka Kuwata^3 **^

**Supplement Figure**

**
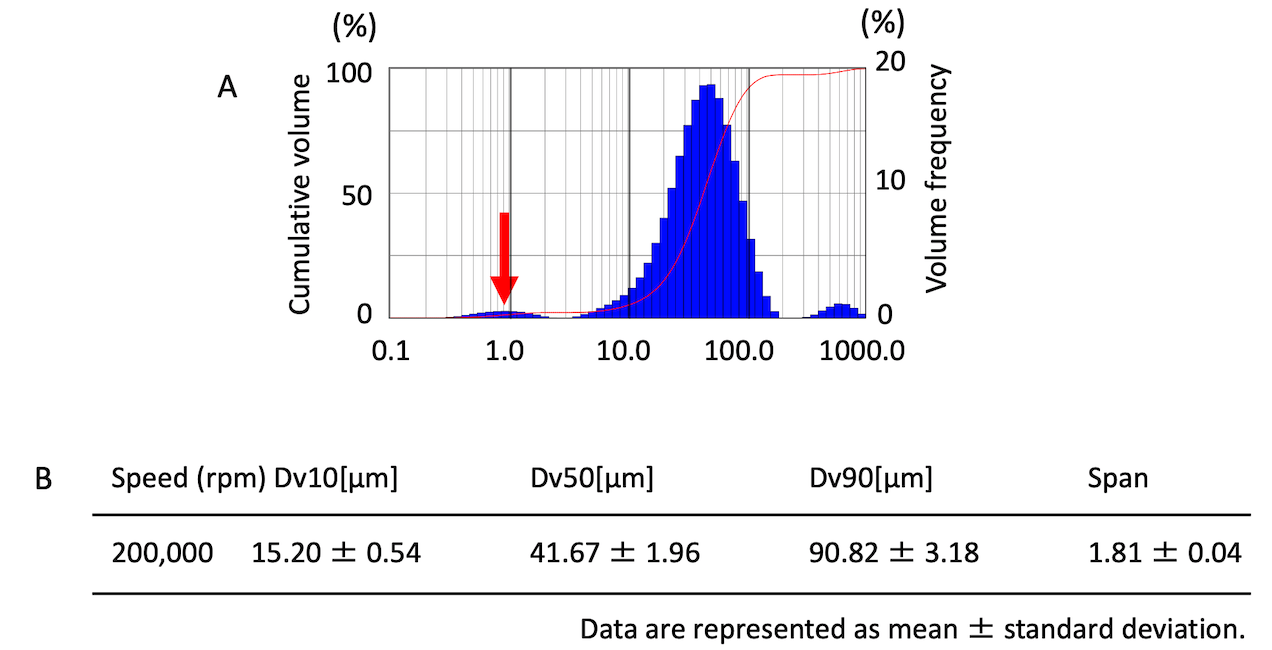
**

Distribution pattern of droplet size emitted from the dental engine at the high speed by the direct-spray measurement. Data from direct spraying is shown (A). Droplet size distribution histogram graph emitted from the dental engine at the speed of 200,000 rpm. The curves in the graphs represent the droplet cumulative volume frequency and the percentage of a particular size of the droplet. The experiment was performed independently at least four times, and the result of one representative experiment is shown. The Dv10, Dv50, Dv90, and Span factors, each of which indicates the 10%, 50% 90% of the cumulative volume of the particles and particle size uniformity, were used (B). Data are represented as mean ± standard deviation.
